# Systematic Reviews: Perhaps “The Answer to Policy Makers’ Prayers”?

**DOI:** 10.1289/ehp.1408599

**Published:** 2014-10-01

**Authors:** Daniel M. Fox, Lisa Bero

**Affiliations:** 1 Milbank Memorial Fund, New York, New York, USA; 2 Department of Clinical Pharmacy, School of Pharmacy, and; 3 Philip R. Lee Institute of Health Policy Studies, School of Medicine, University of California, San Francisco, San Francisco, California, USA

Four articles in this issue demonstrate effective adaptation of the methodology of systematic reviews to environmental health science (Johnson et al. 2014; Koustas et al. 2014; Lam et al. 2014; Woodruff and Sutton 2014). Researchers in many countries and disciplines have participated in developing this methodology for more than a century and have used it to synthesize evidence from primary sources since the 1970s. Systematic reviews currently inform decisions by practitioners and policy makers in clinical medicine and some aspects of public health, as well as in fields such as criminal justice, education, social welfare, addiction services, and international development. Media coverage of systematic reviews has increased steadily since the 1990s. The cumulative result of this attention to systematic reviews among clinicians and policy makers and in the media is that patients and their caregivers accord growing attention to the findings of these reviews.

The variety and rigor of primary sources that are included in systematic reviews has also increased. Systematic reviewing began as a methodology for synthesizing evidence acquired during randomized controlled trials and soon expanded to include data from quasi-experimental research. In recent decades, methodologists have devised ways to include evidence from observational and qualitative research as well. Similarly, methodologists working internationally have established generally accepted rules for detecting systematic bias in primary studies, as well as for grading the quality and strength of the evidence reported in them.

Johnson et al. (2014), Koustas et al. (2014), Lam et al. (2014), and Woodruff and Sutton (2014) offer formidable proof that systematic reviews can inform policy and practice in environmental health. As a group, they offer strong endorsement of the program to encourage the use of systematic reviews to improve policy for environmental health, initiated in 2011 by the Office of Health Assessment and Translation of the National Toxicology Program (Rooney et al. 2014).

The four articles in this issue could have an influence on research, policy, and regulatory practice in environmental health that resembles what four landmark publications achieved in clinical health services between 1988 and 1992 (Fox 2011). The authors and editors of those publications adapted and applied methods from adjacent clinical disciplines and epidemiology to the evaluation of interventions in perinatal care (Fox 2011). This effort evolved to become The Cochrane Collaboration and its library, which currently offers approximately 6,000 systematic reviews (The Cochrane Collaboration 2014). Similarly, the three Reviews and the Commentary in this issue explain methods that participants in the Navigation Guide project adapted from other disciplines—including disciplines that study species other than humans—and then applied them to assess the health risks created by a chemical that abounds in contemporary society.

The landmark collection of systematic reviews of interventions during pregnancy and childbirth published a quarter century ago concluded with four appendices listing “forms of care” that “reduce negative outcomes,” “appear promising,” have “unknown effects which require further evaluation,” or that “should be abandoned” (Chalmers et al. 1989). In contrast, the authors of the articles in this issue of *EHP* used Navigation Guide methodology and concluded that “developmental exposure to PFOA adversely affects human health” (Lam et al. 2014). Many readers of these articles, including regulators, scientists, journalists, and lobbyists, will be eager to learn the results of applying the sections of the Navigation Guide that address factors “brought to bear on recommendations for prevention, including values and preferences, extent of exposures, the availability of safer alternatives, and costs and benefits” (Woodruff and Sutton 2014).

Although this demonstration of the value of the Navigation Guide is incomplete, the work described in these articles exemplifies the intellectual as well as statistical power of systematic reviews. For persons new to the methodology of systematic reviewing, the articles offer compelling examples of why it is more credible than less-rigorous methods of synthesizing research. These examples include criteria for locating, arraying transparently, and selecting relevant studies of primary data; tools for assessing the persistence of systematic biases in these studies; and techniques for evaluating the quality and strength of the evidence selected for inclusion.

Every demonstration of the methods and potential uses of systematic reviews has been incomplete since the first one an author of this editorial (D.M.F.) experienced in Oxford, United Kingdom, in January 1990, when [now Sir] Iain Chalmers showed him the newly published two volumes of *Effective Care in Pregnancy and Childbirth* (Chalmers et al. 1989), the paperback version of it for general readers, and the floppy discs with which he and his collaborators exchanged data in order to update reviews. The story of systematic reviewing is always incomplete because new methods for conducting and applying them constantly emerge. Moreover, reviews must be updated to take account of new relevant evidence.

The story is also incomplete because of the persistence of skepticism about—even opposition to—systematic reviews. Many researchers and policy makers ignore (and some disparage) the logic and hard work of their authors, even when it meets international standards. Most schools that educate for the health professions have not appointed or promoted faculty members whose primary research activity is conducting systematic reviews. In the health sector, commercial organizations in the supply chain for prescription drugs and medical devices, patient advocacy groups they finance, and some associations of physicians continue to oppose policy for covering and paying for particular interventions that are substantially informed by systematic reviews.

Readers of this journal understand the political economy of regulating toxic chemicals in the environment and the extent to which it resembles health policy and regulation. Some of them may, however, be encouraged by the following examples of the use of systematic reviews in the health sector. Most of the states decide which prescription drugs to cover for persons in public programs on the basis of systematic reviews of the comparative effectiveness of drugs in particular classes (Fox 2010a, 2010b). The Patient-Centered Outcomes Research Institute created by the Affordable Care Act (2010) is advancing the methods and uses of reviews in comparing the effectiveness of interventions. Systematic reviews increasingly inform the education and training of clinicians and are the basis of clinical practice guidelines.

The articles by Johnson et al. (2014), Koustas et al. (2014), Lam et al. (2014), and Woodruff and Sutton (2014) are evidence of growing recognition of the potential value of the Navigation Guide as a methodology to assist in regulating chemicals in the environment. A recent historian of the “struggle to define the safety of chemicals” (Vogel 2013) described the Navigation Guide as a pioneering effort “to provide individuals, policy makers, and clinicians with trusted sources of information to assess different recommendations for reducing risk and improving public health.”

The most important lesson from the history of systematic reviews in health services may be how long and intense the struggle to make them routine in environmental health is likely to be. In 1990, one of us (D.M.F.) presented what some observers claim was the first overview of the methods of systematic reviewing to a group of policy makers from several states, beginning with slides of the first page of each of the appendices listing “forms of care” described above. He then asked the audience to assume that he could persuade them that these recommendations were justified by science, and asked how they would judge the information on the slides. “The answer to policy makers’ prayers,” said the chair of appropriations in the House of Representatives of a midwestern state. Everyone nodded agreement. Some years later, a policy maker told the other author how important it is to “develop good taste in evidence” (Jewell 2008). In health services then, as with the Navigation Guide now, we were only at the beginning of the story.
